# Predictive value of hypoxia, metabolism and immune factors for prognosis in hepatocellular carcinoma: a retrospective analysis and multicenter validation study

**DOI:** 10.7150/jca.41983

**Published:** 2020-04-12

**Authors:** Peng Lin, Dong-yue wen, Gang Chen, Yi-wu Dang, Yun He, Hong Yang

**Affiliations:** 1 Department of Medical Ultrasound, The First Affiliated Hospital of Guangxi Medical University, Nanning, Guangxi Zhuang Autonomous Region, China.; 2 Department of Pathology, The First Affiliated Hospital of Guangxi Medical University, Nanning, Guangxi Zhuang Autonomous Region, China.

**Keywords:** hepatocellular carcinoma, tumor microenvironment, prognosis, multi-omics data.

## Abstract

The tumor microenvironment (TME), as a potent and pervasive factor of tumorigenesis and tumor progression, has a profound impact on the clinical outcomes of hepatocellular carcinoma (HCC). A systematic analysis of TME factors in HCC is still lacking and urgently needed. In this retrospective analysis and multicenter validation study, a total of 987 HCC patients with RNA-seq or microarray data and the corresponding clinical information from five cohorts were included. A TME risk score was developed based on five factors (hypoxia, nucleotide, TCA cycle, T helper cells and activated CD8 T cells). We also identified various types of clinical parameters and molecular features associated with the TME risk score. The TME risk factor network depicts close associations among the factors. Our TME risk score could be a practical and reliable predictor that can stratify patients according to distinct clinical outcomes and was validated by integrating five HCC patient cohorts (HR= 2.27, 95% CI: 1.79-2.86, P<0.001). Pan-cancer analysis also suggested that the prognostic signature was an effective prognostic indicator in 9,122 patients across 30 types of cancer. Correlation analysis revealed that the TME risk score was significantly associated with tumor progression-related clinical factors and molecular factors. TME factors are perturbations in HCC patients, and these alterations are vital determinants of both clinical outcomes and biological characteristics. The TME risk score we proposed is valuable for deciphering the molecular characteristics of the TME in HCC and is an effective prognostic predictor for HCC prognosis evaluation.

## Introduction

Tumors are composed of tumor cells and the tumor microenvironment (TME), which represents complex and intimate crosstalk networks across many cellular components and factors, including stromal and immune cells and hypoxic and low pH environments 1. The TME actively participates from the early beginning of carcinogenesis 2. Accordingly, complex cancer TME factors are recognized as common phenomena of tumors and lead to a dilemma in precision medicine. Hypoxia, tumor metabolism and immune cell infiltrates are fundamental to the TME and act as indispensable factors in tumorigenesis and progression 3. Hypoxia activates tumor angiogenesis, modulates cell metabolic processes and leads to the shaping of the TME and distinct tumor characteristics 3. Emerging evidence also indicated that the immune phenotypes of cancers are closely linked to distinct metabolism phenotypes 4. These are multiple TME factors that coexist and interact in diverse cellular pathways and promote tumorigenesis and progression 5.

Liver cancer is the third most lethal malignancy globally 6. Hepatocellular carcinoma (HCC), the most frequent primary liver cancer, is a heterogeneous disease etiologically and biologically 7, 8. When feasible, curative options such as surgery, liver transplantation or radiofrequency ablation represent the treatment of choice, as they offer long-term survival benefits 9. HCC patients in the advanced stage have a dismal prognosis if untreated 10. HCC occurs primarily in patients with underlying chronic liver disease, including infection with hepatitis B virus (HBV) or hepatitis C virus (HCV), alcohol abuse, and several metabolic syndromes 11, 12. HCC is an inflammation-driven disease with potentially chronic liver disease, and molecular differences can underlie the different microenvironment compositions 13. There is marked heterogeneity in malignant cells within and between tumors and diverse TME landscapes 14. Accordingly, the intimate interaction between the hepatic TME and tumor cells should be strongly considered.

Herein, we explore individual differences in terms of TME components affecting the prognosis of HCC. Overall, these findings suggest that the indispensable roles of hypoxia, metabolism and immune cells have great clinical application values. The availability of multi-omics data from The Cancer Genome Atlas (TCGA) project provides unprecedented opportunities to characterize the clinical significance and drug response of hypoxia, metabolism and immune cells in great depth.

## Materials and Methods

### Acquirement of molecular and clinical data

Data from the TCGA project were used as a training cohort to estimate the relationships between TME factors and prognosis. Clinicopathological data and follow-up information were acquired from the TCGA pan-cancer portal (https://gdc.cancer.gov/about-data/publications/pancanatlas). Furthermore, we systematically searched for HCC gene expression datasets that were publicly available and reported clinical outcome information to be used as validation cohorts. In total, we gathered another four cohorts of samples from HCC patients for this study: GSE14520 15, GSE54236 16, GSE76427 17 and LIRI-JP 18. The gene expression matrices of HCC patients in the GSE14520, GSE54236, and GSE76427 datasets were downloaded from the Gene Expression Omnibus (https://www.ncbi.nlm.nih.gov/geo/), and LIRI-JP was downloaded from the International Cancer Genome Consortium (ICGC) database (https://icgc.org/). Only patients with overall survival (OS) times not less than 30 days were included in the present study.

### Estimation of the molecular factor score

To obtain an incisive understanding of the hypoxic, metabolic and immune heterogeneity of HCC, we curated the metagene sets of hypoxia status, seven metabolic pathways and 16 types of immune cells. A 15-gene expression signature (ACOT7, ADM, ALDOA, CDKN3, ENO1, LDHA, MIF, MRPS17, NDRG1, P4HA1, PGAM1, SLC2A1, TPI1, TUBB6 and VEGFA) that has been shown to perform well when classifying hypoxia status was used 19. This gene signature was defined based on gene function and an analysis of *in vivo* co-expression patterns and was highly enriched for hypoxia-regulated pathways. Furthermore, we focused on seven metabolic pathways, including amino acid metabolism, carbohydrate metabolism, integration of energy, lipid metabolism, nucleotide metabolism, tricarboxylic acid cycle (TCA cycle) and vitamin and cofactor metabolism, to systematically analyze the metabolic alterations in HCC 20. For immune cell infiltration estimation, the metagenes of 16 immune cell populations were acquired from a previous study 21.

To quantify the activity changes of these TME factors, the Gene Set Variation Analysis (GSVA) algorithm was implemented 22. The GSVA algorithm produces normalized enrichment scores, which represent the enrichment score in the sample of the analyzed cohort relative to that of other tumors.

### Development of the TME risk score

We further explored the prognostic values of the TME factors to observe their influence on the patients' clinical outcome. A univariate Cox model was used to assess whether these molecular factor enrichment scores were associated with the OS times of HCC patients. Factors with P < 0.05 were considered candidate survival-associated factors and were included in subsequent analyses. Then, a multivariate Cox model was constructed based on the survival-associated factors. The TME risk score was finally calculated by the factor score multiplied by the coefficient from multivariate Cox regression analysis.

### Validation of the TME risk score

The generalization ability of the TME risk score for clinical outcome surveillance should also be analyzed to promote its clinical application. We included four independent datasets to validate the performance of TME risk score. Datasets that provided microarray or RNA-seq data with clinical follow-up information of HCC patients were included. Similarly, OS was the endpoint and patients with OS less than 30 days were removed. GSE14520 dataset consisted of tissues from 242 patients with primary HCCs, who underwent radical resections. The gene expression profiles were determined by Affymetrix GeneChip arrays. GSE54236 dataset consisted of tissues from 78 primary HCC patients who received surgery. The gene expression profiles were determined with the Agilent Microarray. GSE76427 dataset consisted of 95 HCC samples that obtained from patients who underwent radical resection. LIRI-JP dataset consisted of 229 samples with RNA-seq data which belong to Japanese population primarily infected with HBV/HCV. Kaplan-Meier (K-M) plots were generated to observe the difference in OS between HCC patients in the high-risk and low-risk groups based on the median cutoff value. First, the hazard ratio (HR) with 95% confidence interval (CI) of the TME risk score was calculated by univariate Cox analysis to estimate its prognostic value in each cohort. To obtain a solid result, we further conducted a meta-analysis to integrate survival analysis results from the training and validation cohorts. The pooled HR with 95% CI was calculated using STATA software version 14.0 (Stata Corporation, College Station, Texas, USA). An observed HR>1 with a 95% CI that did not cross 1 favored a poor prognosis in HCC patients with a high TME risk score. Either a fixed effect or a random effect model was selected based on the heterogeneity analysis results.

### Integration of the TME risk score and clinical stage

We further observed the relationships between the TME risk score and several clinical parameters. Subgroup analysis by using the “forestplot” package in R software was used to estimate the clinical prognostic value of the TME risk score for different clinical features. Considering that the traditional TNM stage is essential in clinical decision making, we integrated the TME risk score and tumor stage by applying multivariate Cox regression analysis in the TCGA dataset.

For pan-cancer survival analysis, RNA-seq data of 9,122 tumor samples across 30 non-hematologic cancer types with OS not less than 30 days were also downloaded from TCGA pan-cancer portal. TME risk score were calculated for each type cancer based on the formula we proposed.

### Molecular characteristics of the TME risk score

To further identify the biological characteristics related to the TME risk score, we screened TME risk score-positive related genes by Spearman correlation analysis in the TCGA database. Genes with correlation coefficients > 0.3 and P value<0.05 were considered TME risk score-related genes. Then, gene functional enrichment analysis of these genes was performed with the “clusterProfiler” package 23. Items in the biological process (BP), cellular component (CC) and molecular function (MF) categories were included in the analysis.

We further explored the relationships between TME factors and some immunotherapy biomarkers. Tumor mutation burden (TMB) was calculated based on the following formula: TMB= (total count of variants) / (the whole length of exons). We used the varscan2 called variants determined by TCGA to estimate the total count of variants and 38 Mb as the estimate of the exome size. CTLA4, PD-L1, PD1 mRNA expression levels were acquired from RNA-seq profile. T cell receptor (TCR) repertoires are critical for recognition of pathogens and malignant cells and may reflect a robust anti-tumor response. The TCR inference was obtained from previous study 24. Spearman correlation analyses were further used to estimate the relationships between TME factors and some immunotherapy biomarkers.

### Statistical analysis

Survival analyses were conducted based on the “survival” package in R software. The AUC of the time-dependent receiver operating characteristic (ROC) curve was determined by the “survivalROC” package. We compared the TME risk scores between patients in different characteristic groups by using the Wilcoxon rank sum test. A permutation test was used to estimate the correlations between the TME risk score and 10 oncogenic signaling pathways: cell cycle, Hippo signaling, Myc signaling, Notch signaling, Nrf2 signaling, PI3K signaling, RTK-RAS signaling, TGFβ signaling, p53 signaling and β-catenin/Wnt signaling 25.

## Results

### Clinical characteristics of the study population

After removing patients with OS times less than 30 days, 343 HCC patients in the TCGA database were included in the present study. Of these patients, 233 (67.93%) were male and 110 (32.07%) were female. The median age at diagnosis of these patients was 61 years. A total of 238 patients were in stage I and II, and 83 patients were in stage III and IV. In total, 214 and 124 patients were in tumor grade G1-G2 and G3-G4, respectively. Furthermore, 242, 78, 95 and 229 HCC patients with OS times not less than 30 days from four independent cohorts were also included to validate our findings. The distribution and selected demographic characteristics of the HCC patients are summarized in Table 1.

### Prognostic value of TME factors in the training cohort

**Figure 1:**
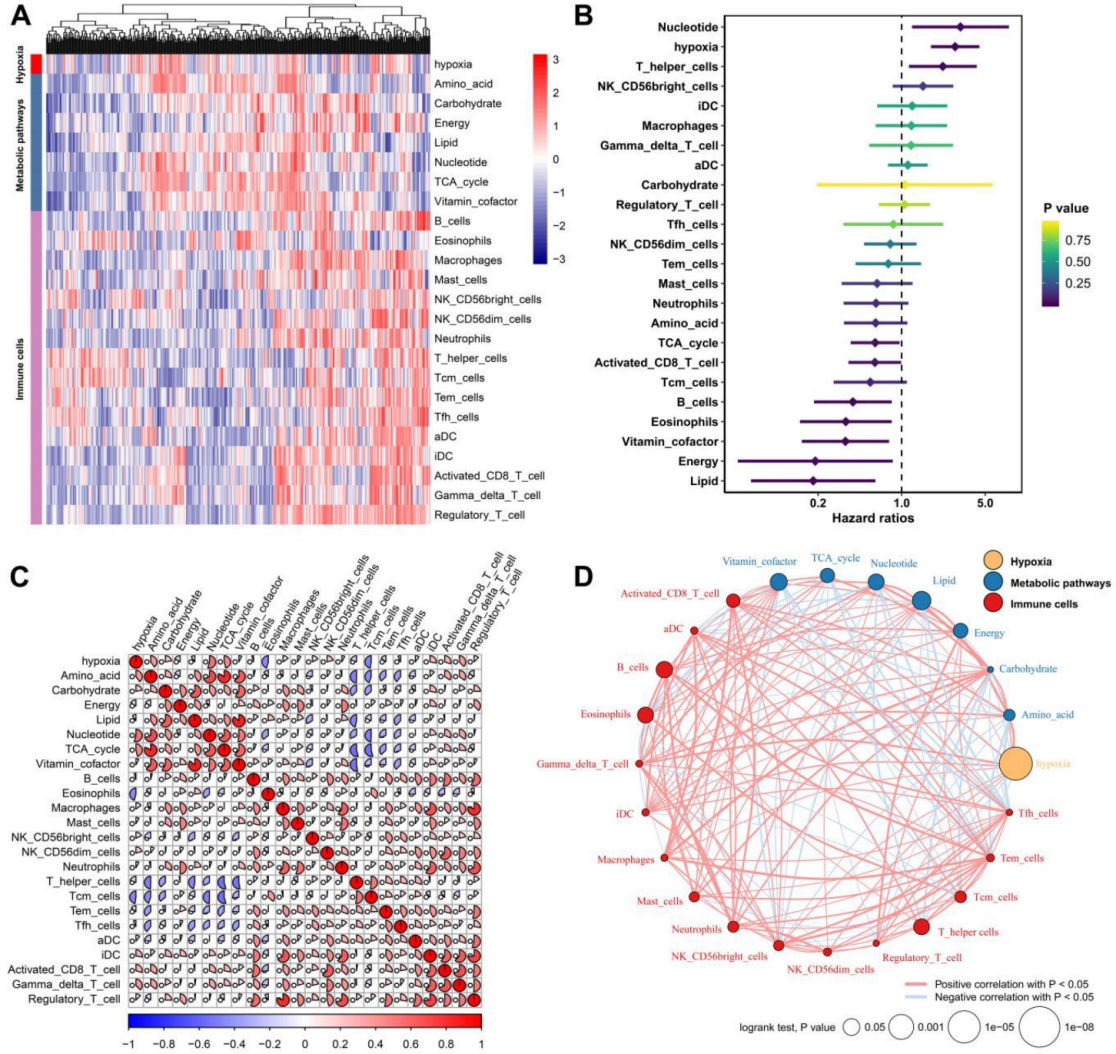
** Landscape of the TME in hepatocellular carcinoma.** (A) Heatmap of hypoxia, metabolic pathways and TME immune cells in HCC patients from the TCGA cohort. (B) Univariate Cox analysis provides relationships between TME factors and overall survival of HCC patients. (C) Correlation relationships between hypoxia, metabolic pathways and immune cells. (D) Factor interaction of the TME factors. The size of each mode represents the survival impact of each TME factor, calculated with the formula log10 (log-rank test P value). The lines connecting TME factors represent cellular interactions. The thickness of the line represents the strength of the correlation estimated by Spearman correlation analysis. A positive correlation is indicated in red, and a negative correlation is indicated in blue.

We defined the hypoxia status, seven metabolic pathways and 16 types of immune cell infiltration patterns of each sample as the relative abundance by using the GSVA algorithm (Figure 1A). By subjecting these TME factors in the TCGA cohort to univariate Cox survival analysis, hypoxia, five metabolic factors (lipid, vitamin cofactor, nucleotide, energy and TCA cycle) and four immune cells (B cells, eosinophils, T helper cells and activated CD8 T cells) that were significantly (P<0.05) correlated with the OS of HCC patients were identified as candidate markers (Figure 1B). These TME factors have close relationships with each other (Figure 1C). Moreover, the TME factor network depicted a comprehensive landscape of the hypoxia status, metabolic pathway and tumor-immune cell interactions and their effects on the OS of HCC patients (Figure 1D). By using Spearman's rank correlation coefficient > 0.3 and P-value < 0.05 for statistical significance, we found that hypoxia was markedly correlated with nucleotide (Cor= 0.544, P<0.001), amino acid (Cor= 0.398, P<0.001) and Gamma_delta_T_cell (Cor=0.380, P<0.001).

**Figure 2:**
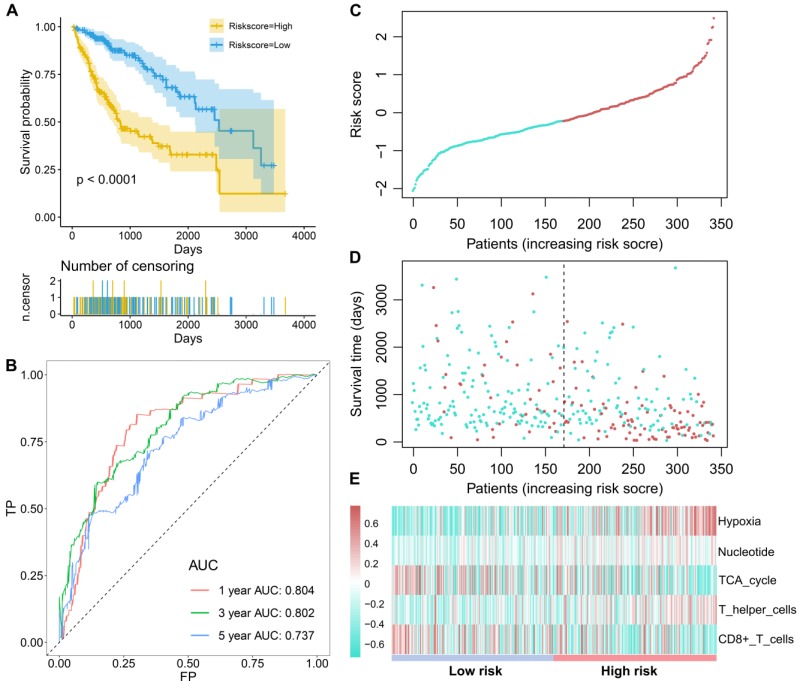
** The survival performance of the TME risk score for HCC patients.** (A), Kaplan-Meier curves of prognostic predictors for HCC. The yellow line indicates the high-risk group, and the blue line indicates the low-risk group. (B) ROC curves of the TME risk score for the 1-, 3-, and 5-year HCC survival prediction. (C) The distribution of the TME risk score for each HCC patient. (D) The relationships between the TME risk score and survival status of HCC patients. (E) Hypoxia, nucleotide and T helper cells are activated in the high-risk group, while TCA cycle and CD8+ T cells are activated in the low-risk group.

Subsequently, these candidate factors were used to perform multivariate Cox stepwise regression analyses. A TME risk score was developed as the following formula: 1.538 * hypoxia +2.496 * nucleotide + (-1.480) * TCA cycle + 0.930 * T helper cells + (-1.148) * activated CD8 T cells. Based on the median TME risk score, HCC patients were divided into high- and low-risk groups. The Kaplan-Meier curve showed that patients in the low-risk group had significantly longer OS times than patients in the high-risk group (P<0.0001, Figure 2A). The time-dependent AUCs were 0.804, 0.802 and 0.737 for 1-, 3- and 5-year OS, respectively (Figure 2B). With increasing TME risk scores, patients suffered a higher risk and inferior OS (Figure 2C-2D). Hypoxia, nucleotide and T helper cells were upregulated in the high-risk group, while the TCA cycle and activated CD8 T cells were downregulated in the high-risk group (Figure 2E).

### Prognostic value of the TME risk score in multicenter validation cohorts

**Figure 3:**
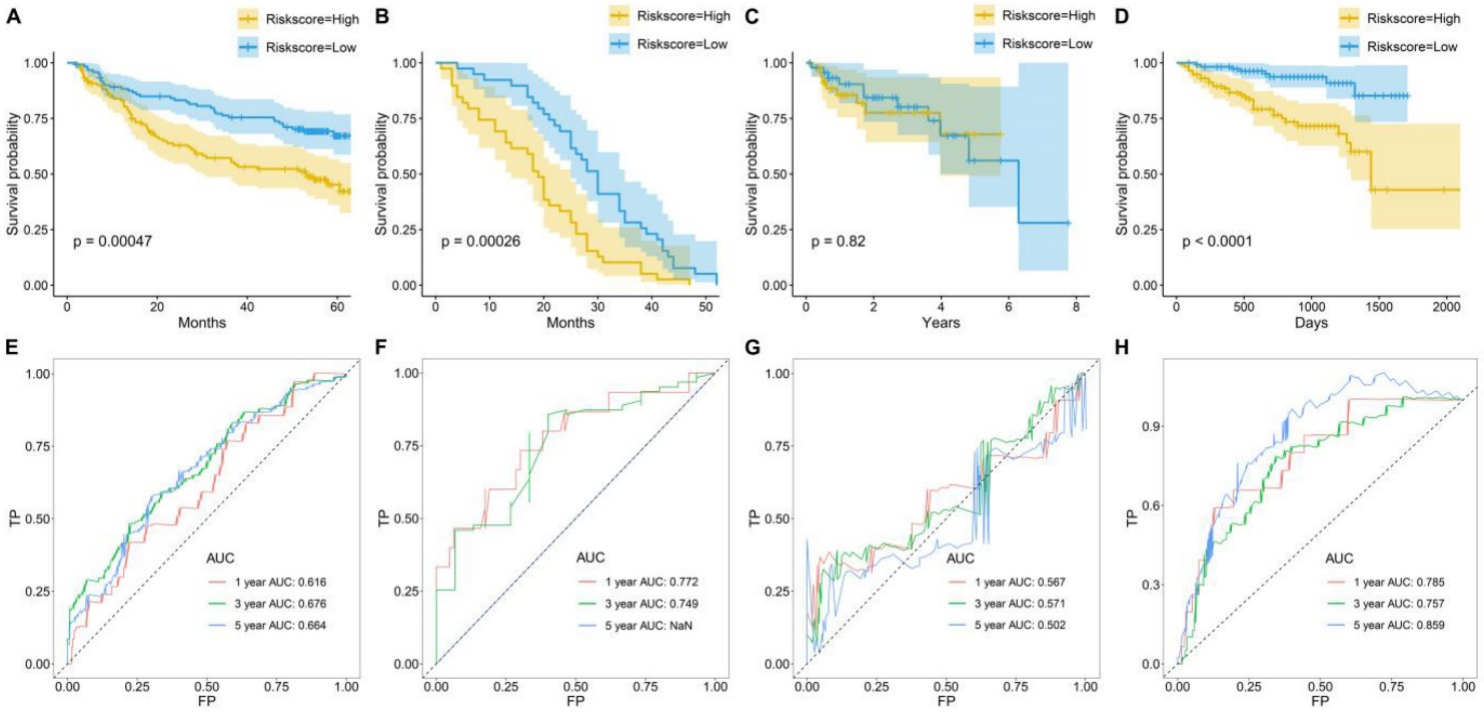
** Survival prediction performance of TME risk score in other four datasets.** Kaplan-Meier curves for the overall survival (OS) of patients from (A) GSE14520, (B) GSE54236, (C) GSE76427, and (D) LIRI-JP. Time-dependent ROC curves for the prediction performance of TME risk score in (E) GSE14520, (F) GSE54236, (G) GSE76427, and (H) LIRI-JP.

To further examine the prognostic significance of the TME risk score in independent cohorts, K-M and ROC analyses were performed in another four independent cohorts. Similarly, patients were separated into high- and low-risk groups based on the median TME risk score. Interestingly, the results showed that the TME score performed well, and patients in the high-risk group suffered a significantly inferior OS compared with those in the low-risk group in GSE14520 (Figure 3A), GSE54236 (Figure 3B) and LIRI-JP (Figure 3D). However, no significance was observed between the high-risk group and the low-risk group in GSE76427 (Figure 3C). In GSE14520, the AUCs were 0.616, 0.676 and 0.664 for 1-, 3-, and 5-year OS (Figure 3E). In GSE54236, the AUCs were 0.772 and 0.749 for 1- and 3-year OS (Figure 3F). In GSE76427, the AUCs were 0.567, 0.571 and 0.502 for 1-, 3-, and 5-year OS (Figure 3G). In LIRI-JP, the AUCs were 0.785, 0.757 and 0.859 for 1-, 3-, and 5-year OS (Figure 3H).

### Combining the TME risk score with Clinical stage

**Figure 4:**
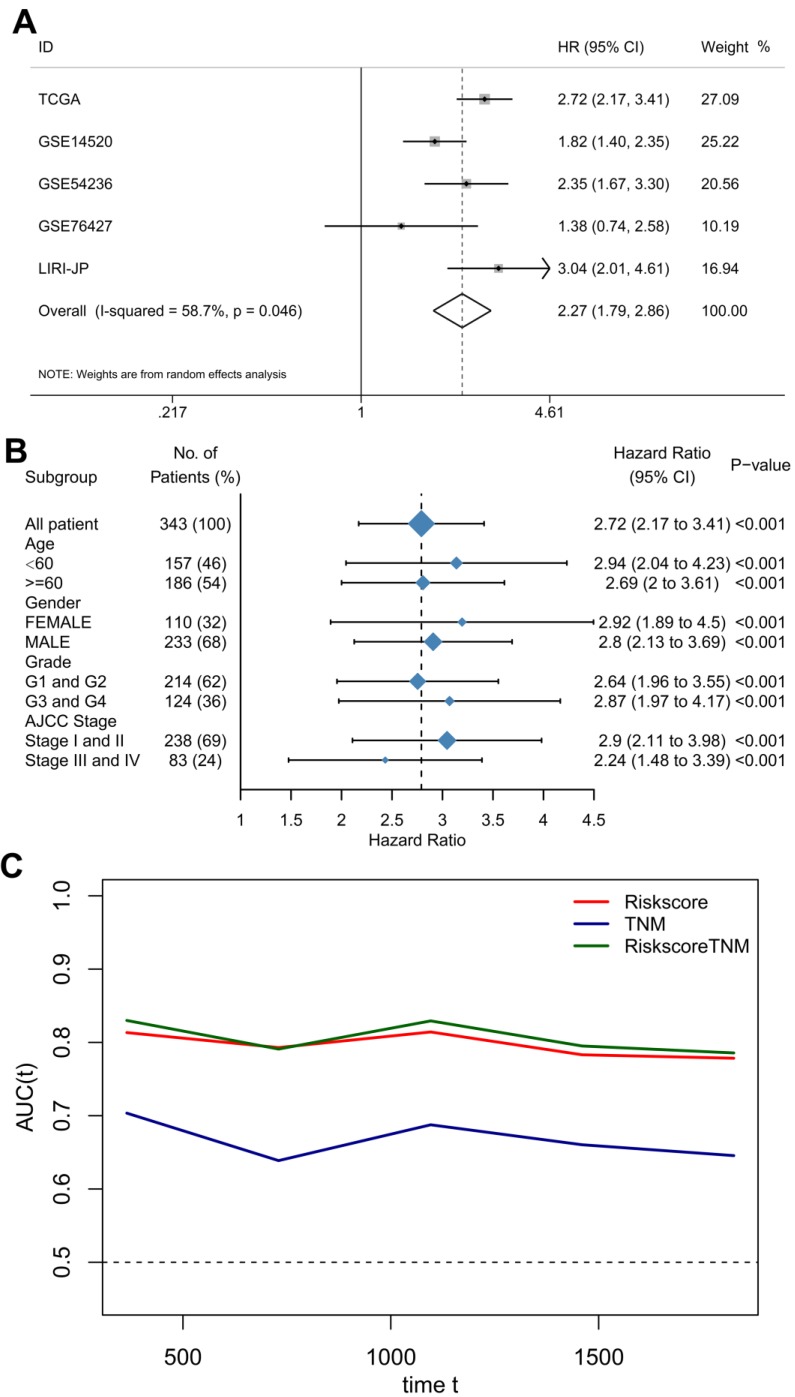
** The TME risk score is a moderate prognostic biomarker.** (A) Forest plot for the association between the TME risk score and overall survival based on five datasets. (B) Subgroup analyses estimating the clinical prognostic value of the TME risk score for different clinical subtypes in TCGA cohort. (C) Time-dependent ROC of TNM, TME risk score and the combination of the two methods.

To provide a robust and comprehensive prognostic value for the TME risk score, we integrated survival analysis from the above five datasets in the form of a meta-analysis. The univariate Cox analysis results of each dataset were collected and generated. The results of the meta-analysis indicated that a high TME risk score was significantly related to a shorter OS (HR= 2.27, 95% CI: 1.79-2.86, Figure 4A). Subgroup analysis based on the TCGA database suggested that the TME risk score was robust in different clinical parameters (Figure 4B).

To further improve accuracy and leverage the prognostic significance of molecular and clinicopathological factors, we combined the TME risk score and tumor stage to fit a Cox proportional hazards model as (0.918 × TME risk score) + (0.347 × tumor stage). However, the AUC, which is the prediction performance assessment value, was not significantly elevated (Figure 4C).

**Figure 5:**
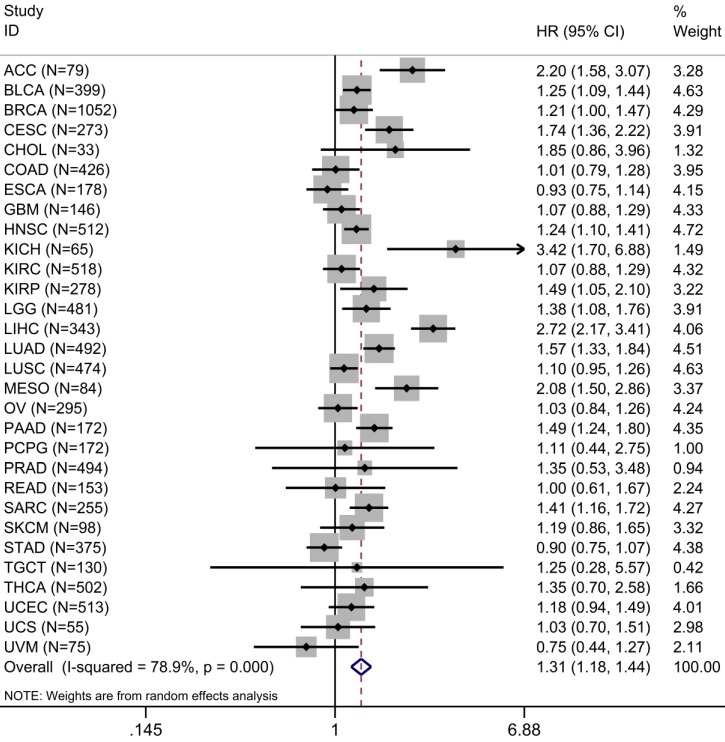
** Prognostic evaluation of TME risk score in pan-cancer patients.** Forest plot visualizing HRs of univariate survival analyses of TME risk score in 30 types of cancer. The random-effects meta-analysis summary of HRs was 1.31 with 95% CI, 1.18-1.44, P <0.001. For a complete list of the TCGA cancer-type abbreviations, please see https:// gdc.cancer.gov/resources-tcga-users/tcga-code-tables/tcgastudy-abbreviations.

A total of 30 types of cancer in 9,122 cases were identified in the survival analysis of TME risk score and OS. Univariate Cox analysis showed that increased TME risk score was significantly associated with lower OS in 12 types of cancers. We integrated HRs for OS in the form of meta-analysis to observe the prognostic value of TME risk score in pan-cancer patients. Increased TME risk score correlated with inferior OS (HR: 1.31, 95% confidence interval: 1.18-1.44, P <0.001) (Figure 5).

### Associations of the TME risk score with clinical parameters and molecular alterations

**Figure 6:**
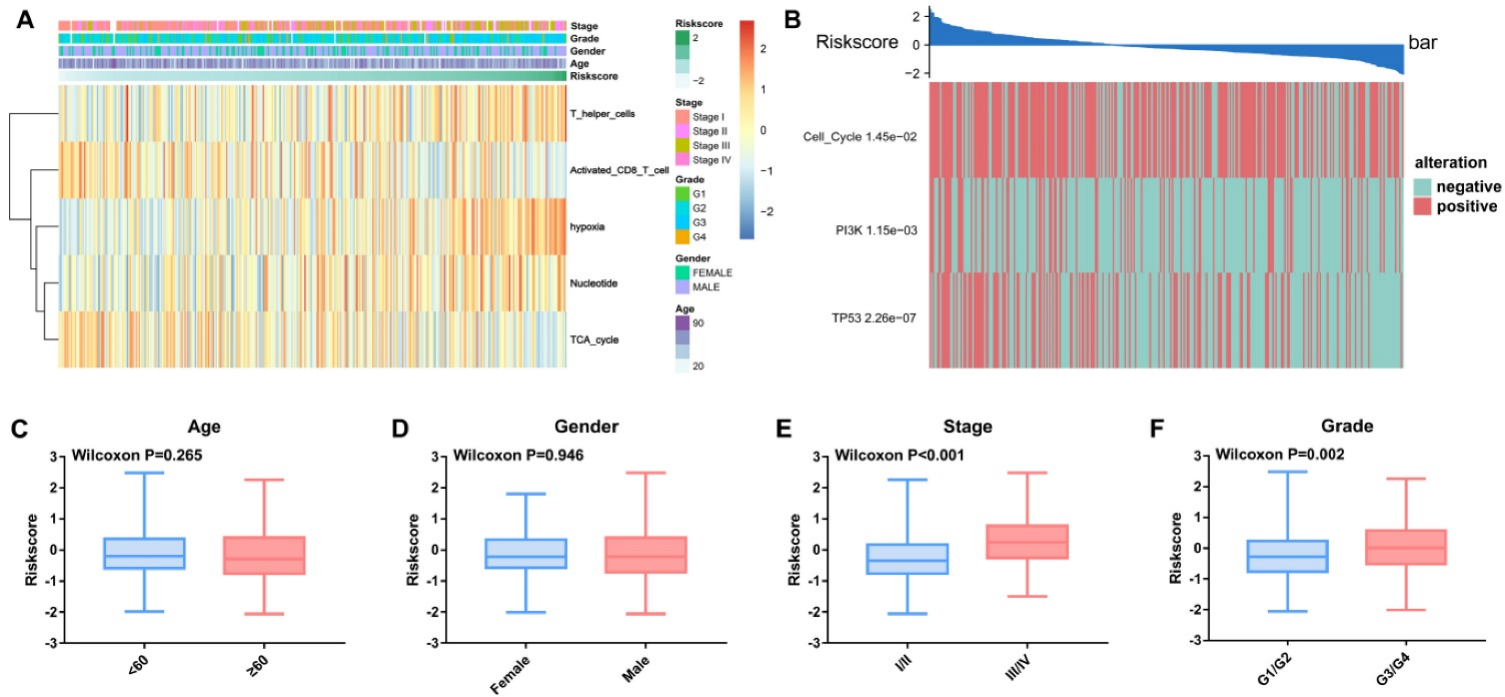
** Relationships between the TME risk score and clinical parameters and oncogenic signaling pathways.** (A) Heatmap of the 343 HCC patients ordered by TME risk score, with annotations associated with each patient. (B) The TME risk score is closely related to genetic alterations in the cell cycle, PI3K and TP53 pathways. The TME risk score was not significantly related to age (C) or sex (D). The TME risk score was significantly related to tumor stage (C) and grade (D).

We observed alterations of oncogenic signaling pathways in patients with different TME risk scores and found that the risk score was significantly correlated with different clinical parameters, including TNM stage and tumor grade (Figure 6A and Figure 6C-F).

Furthermore, three out of ten oncogenic signaling pathways, including the cell cycle, PI3K and p53, were significantly correlated with the TME risk score. The Wilcoxon test validated that the TME risk score was significantly different between patients with pathway alterations and those without alterations (Figure 6B).

### Functional Annotation of the TME risk score

**Figure 7:**
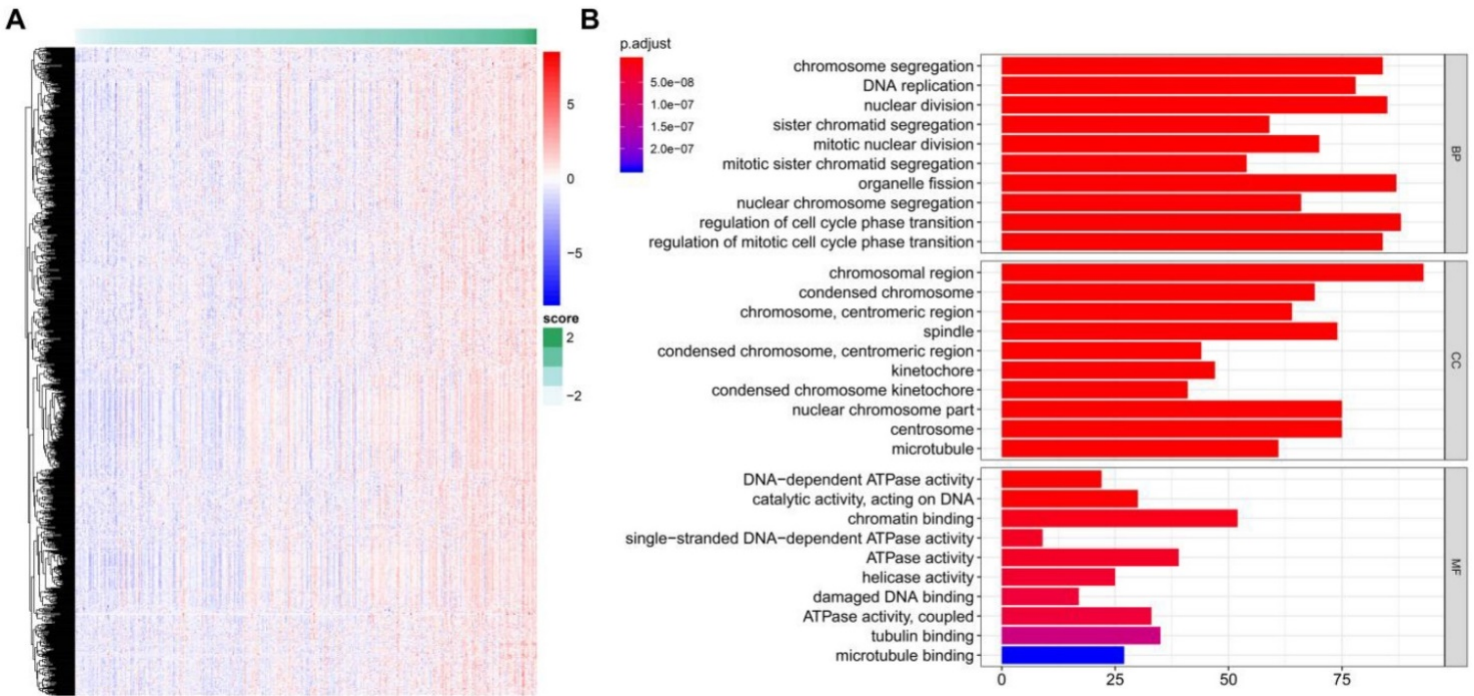
** TME risk score-related genes and functional enrichment analysis.** (A) Identification of genes positively related to the TME risk score. (B) Gene Ontology enrichment analysis of the TME risk score.

A total of 922 TME-related genes were obtained (Figure 7A). Gene functional enrichment analysis revealed that these genes were mainly involved in “chromosome segregation”, “DNA replication” and “nuclear division” of the biological process category; “chromosomal region”, “condensed chromosome” and “chromosome, centromeric region” of the cellular component category; and “DNA-dependent ATPase activity”, “catalytic activity, acting on DNA”, and “chromatin binding” of the molecular function category (Figure 7B).

**Figure 8:**
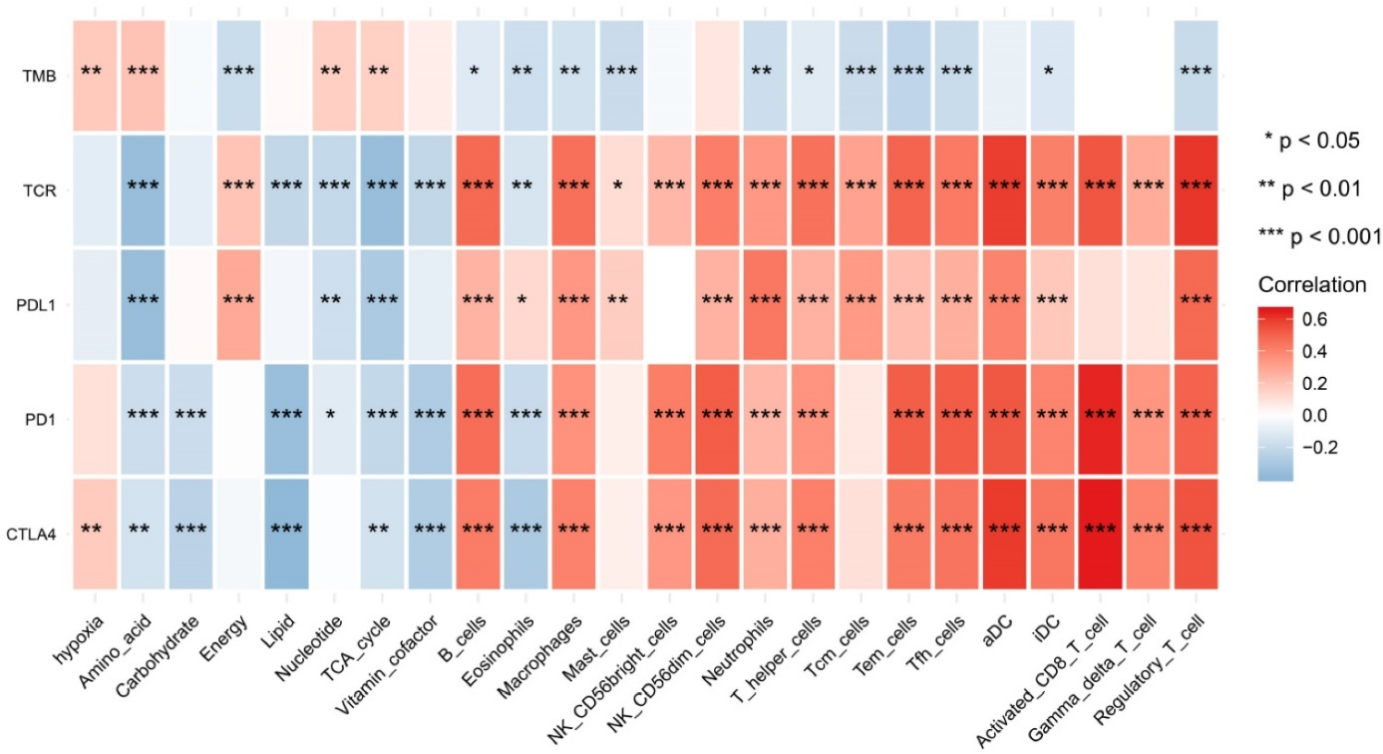
The heatmap of shows correlations between TME factors and immunotherapy biomarkers.

We found that hypoxia status, metabolic pathways and immune cell infiltrates were closely correlated to these immunotherapy biomarkers. The correlation relationships landscape provides a potential regulatory relationships and predictive molecular biomarkers for immunotherapy (Figure 8).

## Discussion

Tumor cells interact with many factors involved in the TME and lead to a unique physiology 26. Mastering the individual TME characteristics offers promising clues for the treatment of many types of cancer 27. This complexity is one of the characteristics of tumors, and it is also the difficulty of clinical tumor treatment. HCC is a disconcerting disease, and even after complete surgical resection, HCC patients still have a high risk of recurrence and death. Reliable prognostic biomarkers are necessary for selecting patients who have high risk in recurrence and death. Considering the vital role of the TME in HCC, prognostic biomarkers identified based on TME components are feasible and have great clinical application potential. Using large-scale datasets, we systematically evaluated the prognostic values of hypoxia status, metabolic pathways and immune cell infiltrates in hepatocellular carcinoma and proposed an TME risk score that precisely evaluated the prognosis. Multi cohorts and pan-cancer data secured the robustness and repeatability of these results. Previously, many prognostic signatures mainly focused on the characteristics of tumor itself, the novel classifier based on tumor microenvironment could provide more valuable information about the prognosis of HCC. As far as we are aware, we are the first group integrated hypoxia status, metabolic pathways and immune cell infiltrates in the field of HCC with large-scale, high-throughput sequencing data to develop precise prognosis model. Furthermore, multi cohorts and pan-cancer data provided novel insights into the robustness and universality of tumor survival prediction. In the era of data exploration, available big data with computational algorithm could contribute to cancer researchers meet the challenges in future.

Hypoxia triggers angiogenesis, rewires cell metabolism and modulates the expression of several immunomodulatory molecules 28, 29. Performing a systematic analysis for the exploration of hypoxia, metabolism and immune cell infiltration is of great significance to understand the molecular characteristics of tumors and guide precision medicine treatment. We first explored the survival significance of hypoxia, metabolism and immune cell infiltrates and their relationships. In general, correlation analyses indicated that there were unique complicated relations among hypoxia, metabolic pathways and immune cell infiltrates. For example, we found that hypoxia was most markedly correlated with nucleotide. These findings suggest that HCC in hypoxic conditions may produce more nucleotides to maintain tumor progression. Nucleotides actively participate in many important cellular processes and are strongly activated in tumor cells and in maintaining the TME 3.

To develop an effective and reliable prognostic classifier for HCC patients, we integrated gene expression profiles from multiple datasets that guaranteed the reliability and generalization ability of the risk score we proposed. The risk score was developed and included hypoxia, nucleotide, TCA cycle, T helper cells and activated CD8 T cells. Hence, it integrated different layers of information of the TME to provide a more precise survival prognosis estimation.

Hypoxia is actively involved in a series of physiological and pathological processes that contribute to carcinogenesis and is significantly correlated with multiple anticancer treatment approaches 30, 31. Nevertheless, hypoxia status remains difficult to evaluate. Although some methods used to diagnose tumor hypoxia have been explored, including oxygen electrode and phosphorescence quenching, photoacoustic tomography and/or endogenous markers of hypoxia, these approaches could not be easily used for large numbers of patient samples^31^. With the advances of high-throughput technology, several studies have documented gene expression signatures that reflect hypoxia status 19, 32, 33. Among them, a 15-gene signature appears to perform the best 19. Many previous studies have validated that hypoxia promotes HCC cell growth, migration and invasion 34-36. Furthermore, a meta-analysis documented that higher levels of hypoxia-inducible factor-1 alpha protein expression indicate a greater possibility of vascular invasion and a poorer clinical outcome in HCC 37. By using the computational algorithm, we also validated the survival value of hypoxia.

Metabolic reprogramming is considered to be closely related to many hallmarks of cancer 38, 39. Tumor cells grow rapidly and absorb nutrients, energy, and biosynthetic compounds, fundamentally changing metabolic activities. Hence, metabolite profiling has gradually been recognized as an informative approach to elucidate tumor heterogeneity 40. Two metabolic factors, nucleotide and the TCA cycle, were also included in the TME risk score. Poor prognosis was significantly associated with the upregulated subtypes of nucleotide but with the downregulated subtypes of the TCA cycle. Many tumor cells undergo a metabolic transition from mitochondria to glycolysis and need rapid proliferation through the truncated TCA cycle 41.

Recently, immunotherapy has substantially changed the therapeutic strategies of many types of tumors, including melanomas 42, lung cancers 43 and HCC 12. A previous meta-analysis validated that high levels of CD8+ tumor-infiltrating lymphocytes had a better prognostic value for OS in HCC patients 44. Another recent meta-analysis that included 3509 patients from 21 observational studies also documented that high levels of intratumoral CD8+ cells were correlated with better OS and disease-free survival 44. T helper cells mainly participate in tumor immunology and are subdivided into subsets, including T1, T2, and T17 cells 45. Th1 cells are associated with a good prognosis in patients with HCC, whereas Th2 and T17 cells are related to tumor growth or metastasis 46, 47. The TME risk score was further validated by another four datasets. Meta-analysis provided a comprehensive view and validated its moderate performance.

One of the main limitations of the present study is its retrospective nature, although we used four independent cohorts to rigorously validate the performance of the TME risk score. Furthermore, the complex nature of HCC implies that not all TME-related factors were included, and further exploration will consider diverse molecular characteristics, which could provide a more precise molecular landscape of HCC.

In conclusion, we developed a TME risk score based on hypoxia status, metabolic pathways and immune cell infiltrates that is a promising prognostic biomarker in HCC. Future explorations are needed to further validate its accuracy for survival prediction and use in the individualized management of HCC.

## Abbrevistion

Tumor microenvironment (TME); Hepatocellular carcinoma (HCC); hepatitis B virus (HBV); hepatitis C virus (HCV); The Cancer Genome Atlas (TCGA); overall survival (OS); International Cancer Genome Consortium (ICGC); tricarboxylic acid cycle (TCA cycle); Gene Set Variation Analysis (GSVA); Kaplan-Meier (K-M); hazard ratio (HR); confidence interval (CI); biological process (BP); cellular component (CC); molecular function (MF); receiver operating characteristic (ROC).

## Figures and Tables

**Table 1:** Demographic and clinical characteristics included in the present study.

Variables n	TCGA (343)	GSE14520 (242)	GSE54236 (78)	GSE76427 (95)	LIRI-JP (229)
Age (median)					
<60	157	192	-	41	44
≥60	186	50	-	54	185
Gender					
Male	233	211	61	82	168
Female	110	31	17	13	61
Tumor grade					
G1-G2	214	-	-	-	-
G3-G4	124	-	-	-	-
NA	5	-	-	-	-
Tumor stage					
I-II	238	174	-	72	141
III-IV	83	51	-	22	88
NA	22	17	-	1	0
